# Effect of newly developed scissors-attached micro-forceps on the recipient clamp time and occurrence of anastomotic site infarction after bypass surgery for moyamoya disease

**DOI:** 10.3389/fneur.2023.1269400

**Published:** 2023-10-06

**Authors:** Munetaka Yomo, Ryuhei Kitai, Hiroyuki Tada, Makoto Isozaki, Yoshifumi Higashino, Ken Matsuda, Takahiro Yamauchi, Ayumi Akazawa, Satoshi Kawajri, Mizuki Oiwa, Shintaro Yamada, Tadahiro Tsubota, Akifumi Watanabe, Hidehiko Okazawa, Yasushi Kiyono, Hidetaka Arishma, Kenichiro Kikuta

**Affiliations:** ^1^Division of Medicine, Department of Neurosurgery, Faculty of Medical Sciences, University of Fukui, Fukui, Japan; ^2^Department of Neurosurgery, Kaga Medical Center, Kaga, Japan; ^3^Technology Development Section, Charmant Co., Ltd., Sabae, Japan; ^4^Biomedical Imaging Research Center, University of Fukui, Fukui, Japan; ^5^Life Science Innovation Center, University of Fukui, Fukui, Japan

**Keywords:** moyamoya disease, STA-MCA anastomosis, clamp time, ischemia, anastomotic site, micro-forceps

## Abstract

**Introduction:**

This study aimed to examine the effect of newly developed scissors-attached micro-forceps in superficial temporal artery-to-middle cerebral artery (STA-MCA) anastomosis for moyamoya disease (MMD).

**Materials and methods:**

Of 179 consecutive STA-MCA anastomoses on 95 hemispheres of 71 MMD patients at the University of Fukui Hospital between 2009 and 2023, 49 anastomoses on 26 hemispheres of 21 patients were enrolled in this retrospective cohort clinical trial intraoperative indocyanine green video-angiography did not demonstrate bypass patency in three anastomoses in two patients who were excluded. Twenty-one anastomosis in 19 hemispheres of 16 patients were performed using the conventional micro-forceps (conventional group, CG), and 25 anastomoses in 22 hemispheres of 19 patients were performed using scissors-attached micro-forceps (scissors group, SG). A small infarction near the anastomotic site detected using postoperative diffusion-weighted imaging was defined as anastomotic site infarction (ASI). Factors affecting the occurrence of ASI were examined by univariate, logistic regression, and receiver operating curve (ROC) analysis.

**Results:**

There were no significant differences in clinical parameters such as age, sex, number of sacrificed branches, number of sacrificed large branches, and number of sutures between the CG and SG. However, the clamp time and occurrence of ASI were significantly lower in the SG than in the CG. Logistic regression analysis revealed that the clamp time was the only significant factor predicting the occurrence of ASI. A receiver operating curve analysis also revealed that the clamp time significantly predicted the occurrence of ASI (area under the curve, 0.875; cutoff value, 33.2 min).

**Conclusion:**

The newly developed scissors-attached micro-forceps could significantly reduce the clamp time and occurrence of ASI in STA-MCA anastomosis for MMD.

## 1. Introduction

Superficial temporal artery-to-middle cerebral artery (STA-MCA) anastomosis was first reported by Yasargil (1). In Japan, it has been applied mainly for the treatment of moyamoya disease (MMD) (2), which is a progressive steno-occlusive disease of the terminal portion of the bilateral internal carotid arteries (ICAs) with the development of moyamoya vessels as collateral channels (3). A previous study combined direct bypass (STA-MCA anastomosis) and indirect bypass (encephalo-duro-arterio-synangiosis) for the treatment of MMD (4). A recent meta-analysis showed that combined and direct bypasses significantly benefited patients with MMD suffering from late stroke and hemorrhage compared to indirect bypass (5). Although STA-MCA anastomosis requires clamping of the MCA for some time, few reports have considered the clamp time of STA-MCA anastomosis for MMD (6).

Recent advances in magnetic resonance (MR) technology, including 3 Tesla MR imaging (3T MRI), allow the detection of asymptomatic small ischemic lesions after STA-MCA anastomosis by diffusion-weighted imaging (DWI) (7). In this study, we examined the effect of newly developed scissors-attached micro-forceps on the clamp time in STA-MCA anastomosis for MMD and examined the effect of the clamp time on the occurrence of asymptomatic small ischemic lesions after surgery.

## 2. Materials and methods

### 2.1. Enrollment of patients

**Figure 1:**
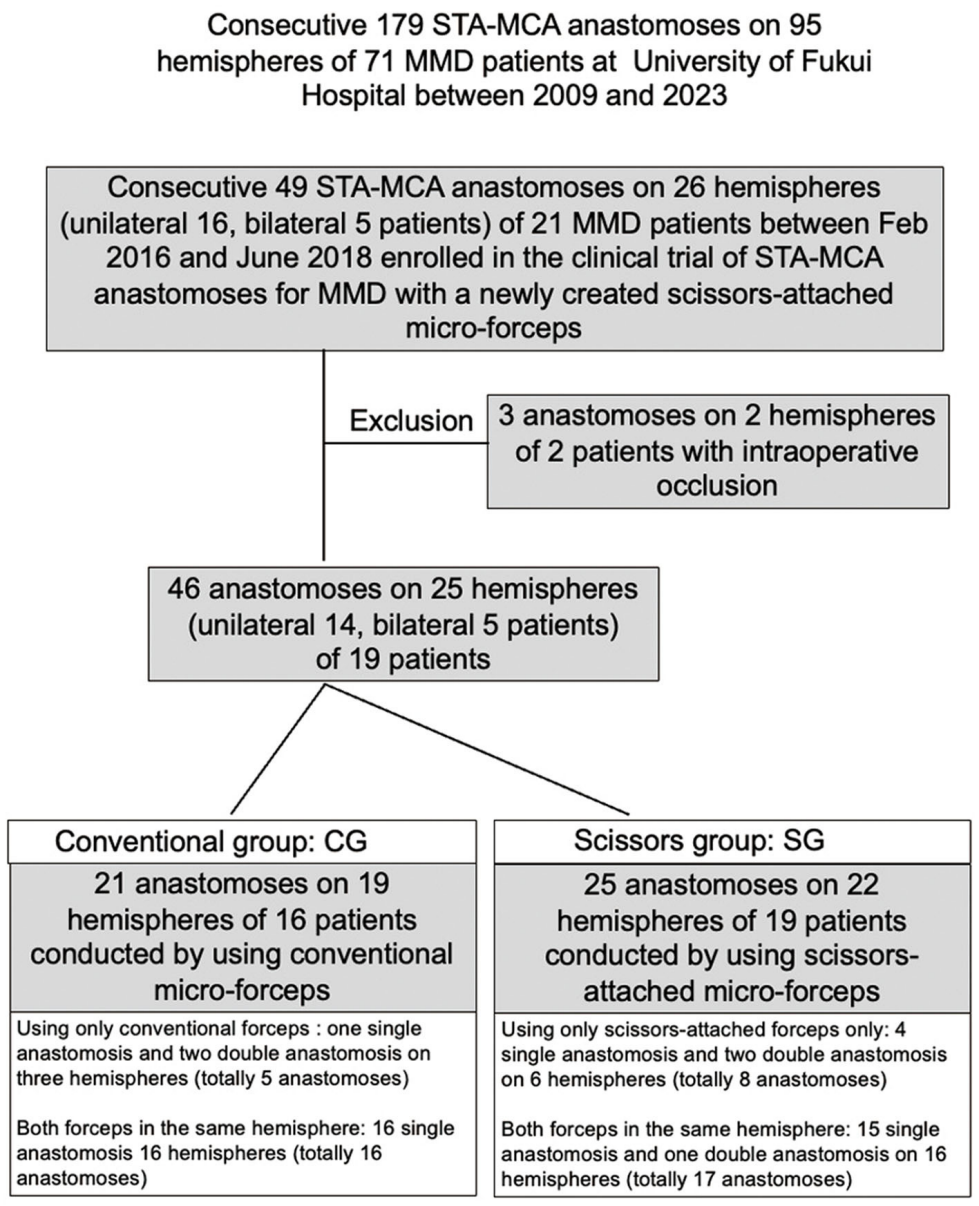
Inclusion of the patients in this clinical trial.

We started direct bypass treatment for MMD at the University of Fukui Hospital in September 2009. Of 179 consecutive STA-MCA anastomoses on 95 hemispheres of 71 MMD patients at the University of Fukui Hospital between 2009 and 2023, 49 anastomoses on 26 hemispheres of 21 patients were enrolled in this clinical trial. Three anastomoses on two hemispheres in two patients were excluded because bypass occlusion was confirmed using intraoperative indocyanine green (ICG) video-angiography (VA). Two anastomoses on one hemisphere in one patient were performed using scissor-attached micro-forceps and were occluded probably due to the thrombus formation in the donor artery derived from the injury of STA during the harvest. Although the reconstruction of STA was performed by end-to-end anastomosis, repeated anastomosis under the administration of anti-platelet drugs could not relieve the occlusion. Another anastomosis in another patient conducted by using conventional micro-forceps was occluded probably due to the complexity of anastomosis owing to the discrepancy of diameters between the donor artery (2 mm) and the recipient artery (0.5 mm). Ultimately, 46 anastomoses on 25 hemispheres in 19 patients were included in this study. Anastomosis in the unilateral hemisphere was performed in 14 patients and on both hemispheres in 6 patients. Among 46 anastomoses on 25 hemispheres in this study, single, double, and triple anastomoses underwent 5, 19, and 1 hemispheres, respectively. While both conventional micro-forceps and newly created scissors-attached micro-forceps were used for double or triple bypass in the same operation on 17 hemispheres, only one of them was used for single anastomosis on five hemispheres in five patients and double anastomosis on three hemispheres in three patients. Accordingly, 21 anastomoses on 19 hemispheres of 16 patients were performed using the conventional micro-forceps (conventional group: CG), and 25 anastomoses on 22 hemispheres of 19 patients were performed using the scissors-attached micro-forceps (scissors group: SG; Figure 1). This study was approved by the Ethics Committee of our institute (No. 20150155), and informed consent was obtained from all patients.

### 2.2. The newly developed micro-forceps

**Figure 2:**
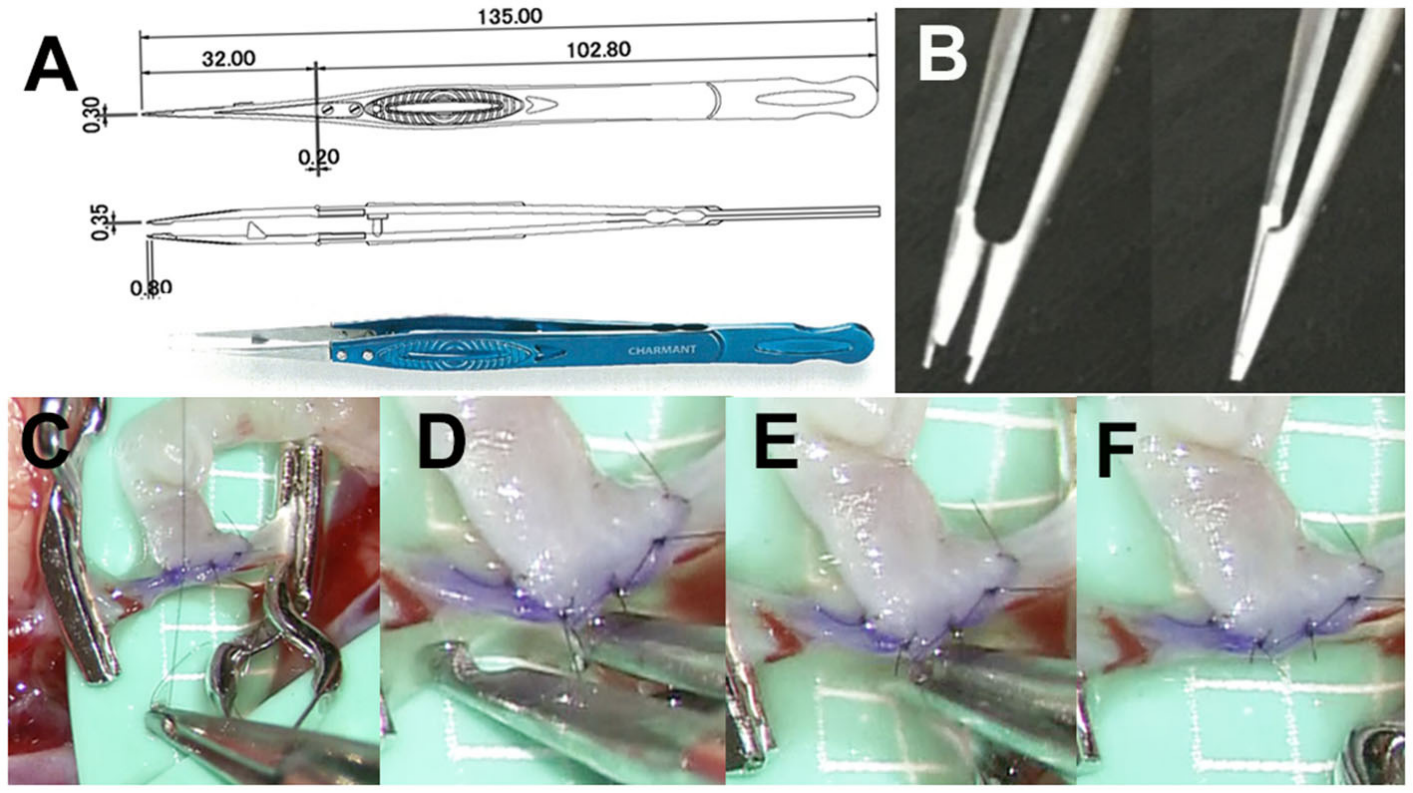
**(A)** Pair of micro-forceps is 135 mm long and consists of three parts: body, head, and tip (Charmant Co., Ltd., Fukui, Japan, www.charmant.co.jp), which are made of Ti15V3Al3Cr3Sn, stainless steel (SUS304), and aged stainless steel (SUS20J-2), respectively. Each part is fixed and connected by screws made of stainless steel (SUS304). **(B)** The tip is 0.35 mm wide and 0.8 mm long. There is a small pair of scissors which is 0.8 mm from the tip for cutting ligatures. **(C)** This small-shaped tip design makes it possible to work with 11-0 ligatures and needles smoothly. **(D–F)** The micro-forceps allow the surgeon suture, tie, and cut a ligature without exchanging instruments, thus reducing the clamp time.

The newly developed micro-forceps are 135 mm long and consist of three parts: body, head, and tip (Charmant Co., Ltd., Fukui, Japan, www.charmant.co.jp), which were made of Ti15V3Al3Cr3Sn, stainless steel (SUS304), and aged stainless steel (SUS20J-2), respectively. Each part is fixed and connected using stainless steel screws (SUS304; Figure 2A). The tip is 0.35 mm wide and 0.8 mm long. A small pair of scissors is placed at a distance of 0.8 mm from the tip for cutting ligatures (Figure 2B). This small-shaped tip design makes it possible to deal smoothly with 11-0 ligatures and needles (Figure 2C). The micro-forceps allow the surgeon to suture, tie (Figure 2C), and cut a ligature without exchanging instruments (Figures 2D–F), thus reducing the clamp time.

### 2.3. Operative procedures and perioperative management

A large U-shaped scalp incision was made around the ears. After reflection of the scalp and temporal muscles, a frontotemporal craniotomy was performed to expose the frontal lobe, Sylvian fissure, and temporal lobe. In most cases, we performed double anastomosis using the parietal and frontal STAs. The supra-Sylvian M4 portion of the MCA and infra-Sylvian M4 portion immediately beside the Sylvian vein were selected as the recipient arteries. Several lateral branches of the recipient artery were coagulated and sacrificed to obtain a 10 mm long branch-free recipient. After clamping the recipient artery, a circular arteriotomy was performed at its roof.

The STA-MCA anastomosis was performed using 8–10 sutures with 11-0 ligatures. One anastomosis was performed using the conventional micro-forceps, and the other was performed using the scissors-attached micro-forceps. When harvesting two branches of the STA was difficult, a single anastomosis was performed. In this case, the micro-forceps used for the anastomosis were randomly selected by the surgeon.

ICG-VA was performed in all the cases. Occlusion of three anastomoses in two patients was confirmed using ICG-VA at the final stage of the operation. After the STA-MCA anastomosis, the temporal lobe was covered with the temporal muscle using encephalo-myo-synangiosis. During the procedure, the systolic blood pressure was maintained between 100 and 140 mmHg, and the PaCO_2_ was strictly maintained between 35 and 45 mmHg. The surgery was performed by KK and four other neurosurgeons.

### 2.4. 3T MRI study

MR studies were performed before surgery and within 1 week after surgery. Images were obtained using a 3T MR scanner (Signa 3-HD, General Electric, USA) with axial DWI (spin echo EPI, TR/TE = 6,300/64.4 ms, bandwidth = ±250 kHz, FOV = 240, slice thickness/gap = 5/1 mm, matrix = 128 × 256, 2NEX), axial FLAIR sequences (fast spin echo, TR/TE/TI = 10,000/120/2,450 ms, bandwidth = ±35.71 kHz, FOV = 240 mm, slice thickness/gap = 5/1 mm, matrix = 320 × 192, 1NEX), and three-dimensional time-of-flight MR angiography [(3D TOF MRA) (3D SPGR, TR/TE = 22/3.5 ms, flip angle = 18°, band width = ±25 kHz, FOV = 180 mm, matrix = 320 × 192, slice thickness/gap = 1.2/−0.6 mm (ZIP2), number of slabs = 3 (location per slab = 28; overlap, five slices), 1NEX, ASSET Factor = 1.5)].

### 2.5. Anastomotic site infarction and symptomatic major infarction after bypass surgery

**Figure 3:**
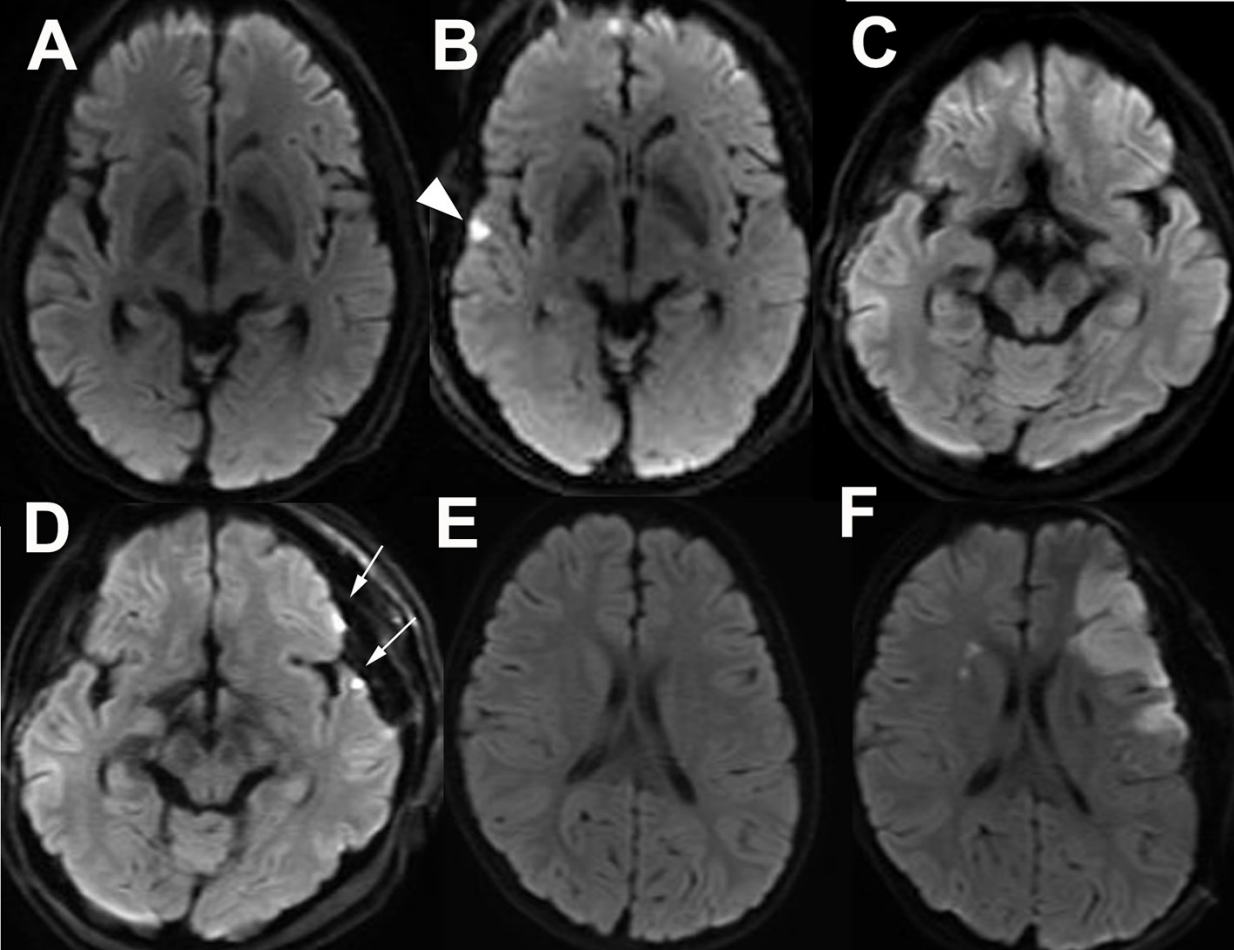
Asymptomatic small infarct lesion at the frontal and or temporal lobes adjacent to the Sylvian fissure detected by postoperative diffusion-weighted imaging (DWI) is defined as an anastomotic site infarction (ASI). Preoperative **(A)** and postoperative **(B)** DWI of a patient with temporal ASI (arrow head). Preoperative **(C)** and postoperative **(D)** DWI of a patient with frontal and temporal ASIs (arrows). Preoperative **(E)** and postoperative **(F)** DWI of a patient with major postoperative infarction at the affected side is distinguished from ASI.

Anastomotic site infarction (ASI) was defined as an asymptomatic DWI-hyperintense lesion with a diameter <5 mm on the cortex near the Sylvian fissure detected by postoperative 3T MRI. An ASI at the temporal lobe was determined as a small ischemic lesion related to the bypass to the infra-Sylvian MCA (preoperative MRI: Figure 3A, postoperative MRI: Figure 3B). ASIs at the frontal and temporal lobes were determined as ischemic lesions related to the bypass to both the supra- and infra-Sylvian MCAs (preoperative MRI: Figure 3C, postoperative MRI: Figure 3D). Symptomatic large DWI-positive lesions or lesions in the contralateral brain were identified as major infarctions related to bypass surgery for MMD and were distinguished from ASIs (preoperative MRI: Figure 3E, postoperative MRI: Figure 3F).

### 2.6. Statistical analysis

A univariate analysis was performed with Pearson's chi-squared test, Fisher's exact test for categorical variables, or using the Mann–Whitney *U*-test for numeric variables. Forward and backward stepwise logistic regression analyses with the Akaike information criterion (AIC) were carried out to determine the associations of potential factors and the occurrence of ASI. The cutoff values for the receiver operating characteristic (ROC) analysis using the area under the curve (AUC) were calculated using Benis' method. All statistical analyses were performed using the JMP software (Version 10, SAS Institute Inc., Cary, NC, USA) and R (R Foundation for Statistical Computing, Vienna, Austria), with an error probability of <0.05.

## 3. Results

### 3.1. Clinical parameters of the CG and SG

There were 16 patients [(mean age, 35.4 ± 18.4 years; male-to-female ratio (M:F) = 3:13)] in the CG (21 anastomoses cases on 19 hemispheres) and 19 (mean age 33.0 ± 20.1 years, M:F = 2:17) in the SG (25 anastomoses cases on 22 hemispheres). There were 10 patients with preprocedural in the CG group and 11 in the SG group, respectively. There was one patient recent ischemic stroke in each group. There were 12, 3, and 1 patients with a preoperative modified Rankin score (preop mRS) of 0, 1, and 2, respectively in CG (mean 0.31±0.60). There were 13, 3, 2, and 1 patients with preop mRS of 0, 1, 2, and 3, respectively, in SG (mean 0.52 ± 0.90). There were 2, 13, and 1 patients with the preoperative Suzuki stage of 2, 3, and 4, respectively, in CG (mean 2.94 ± 0.44). There were 6, 11, 1, and 1 patients with the preoperative Suzuki stage of 2, 3, 4, and 5, respectively, in SG (mean 2.84 ± 0.76) (8). Regarding the outcome of bypass function, there were 7, 7, and 2 patients with Matsushima grades A, B, and C, respectively, determined by postoperative angiography at 3 months after the last surgery in CG. There were 9, 8, and 2 patients with Matsushima grades A, B, and C, respectively, in SG. The mean follow-up period of CG and SG were 67.0 ± 18.4 and 69.4 ± 15.9 months, respectively (9). There were 12 and 4 patients with mRS at the final follow-up of 0 and 1, respectively, in CG (mean 0.25 ± 0.45). There were 13, 4, and 1 patients with preop mRS of 0, 1, 2, and 3, respectively, in SG (mean 0.47 ± 0.84). There was no significant difference in the number of patients, number of anastomoses, number of males, mean age, the presence of preprocedural infarction, recent ischemic stroke, preop mRS, Suzuki stage, Matsushima grade of postoperative angiography, mean follow-up period, and mRS at the final-follow up between the two groups. In a patient-oriented comparison, the mean number of sacrificed lateral branches of the recipient artery in CG (21 anastomose) and SG (25 anastomose) were 2.4 ± 1.1 and 2.9 ± 2.2, respectively. The mean number of sacrificed lateral branches with a diameter larger than 200 mm in CG and SG were 0.14 ± 0.36 and 0.2 ± 0.58, respectively. The mean number of sutures was 10.0 ± 1.2 and 10.0 ± 1.8, respectively. There was also no significant difference in the number of sacrificed lateral branches, the number of sacrificed lateral branches with a diameter larger than 200 μm, and the number of sutures between the two groups (Table 1).

**Table 1:** Characteristics of the group that underwent anastomoses using conventional forceps (Conventional group: CG) and that which underwent anastomoses using scissors-attached forceps (Scissors group: CG).

		**Conventional group (CG)**	**Scissors group (SG)**	***p*-value**
No. of anastomose		21	25	1.000
No. of hemispheres		19	22	1.000
No. of patients		16	19	1.000
No. of male		3	2	0.6418
Mean age (years)		35.4 ± 18.4	33.0 ± 20.1	0.9207
Preprocedural infarction		10	11	1.000
Recent ischemic stroke		1	1	1.000
Preoperative mRS		0.31 ± 0.60	0.52 ± 0.90	0.5734
Suzuki stage	2	2	6	0.3942
	3	13	11	
	4	1	1	
	5	0	1	
	mean	2.94 ± 0.44	2.84 ± 0.76	
No. of sacrificed branches		2.4 ± 1.1	2.9 ± 2.2	0.7249
No. of sacrificed branches with the diameter larger than 200 μm		0.14 ± 0.36	0.2 ± 0.58	0.9099
No. of sutures		10.0 ± 1.2	10.0 ± 1.8	0.5306
Matsushima grade at postoperative angiography	A	7	9	0.9705
	B	7	8	
	C	2	2	
Mean follow-up period (months)		71.1 ± 12.3	69.4 ± 15.9	0.8813
mRS at final follow-up		0.25 ± 0.45	0.47 ± 0.84	0.5573

### 3.2. Effect of scissors-attached micro-forceps on the clamp time, clamp time per suture, and occurrence of ASIs

**Figure 4:**
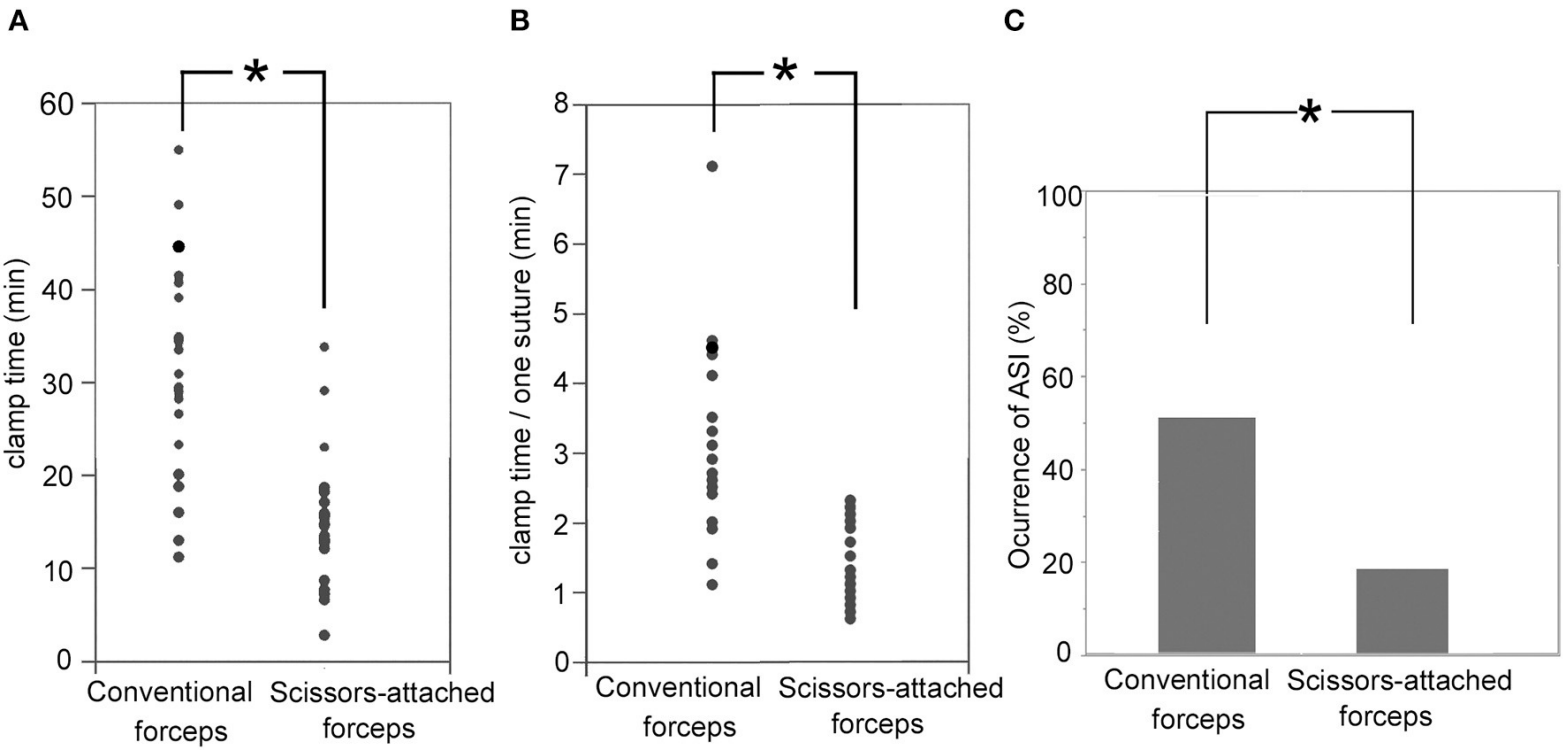
**(A)** Clamp time of the recipient artery regarding the SG (24.2 ± 6.9 min) was significantly shorter than CG (41.0 ± 11.6 min; *p* < 0.001). **(B)** The clamp time per suture in the SG (2.4 ± 0.5 min) was significantly shorter that in the CG (4.2 ± 1.4 min; *p* < 0.001). **(C)** Occurrence rates of ASI in the SG (16%) were significantly smaller than that in the CG (67%; *p* = 0.0312). ^*^Indicates statistically significant.

The clamp time of the recipient artery regarding the CG and SG was 41.0 ± 11.6 min and 24.2 ± 6.9 min, respectively. The clamp time in the SG was significantly shorter than that in the CG (*p* < 0.001; Figure 4A). Furthermore, the clamp time per suture in the CG and SG was 4.2 ± 1.4 and 2.4 ± 0.5 min, respectively. The clamp time per suture was significantly shorter in the SG than in the CG (*p* < 0.001; Figure 4B). The occurrence rates of ASI in the CG and SG were 67 and 16%, respectively. The occurrence of ASIs in CG and SG was 52.3 and 20.0%, respectively. The occurrence of ASIs was more significant in the CG than in the SG (*p* = 0.0312; Figure 4C).

### 3.3. Logistic regression analysis regarding the prediction of ASI occurrence

Forward and backward stepwise logistic regression analyses with AIC revealed the occurrence of ASI were predicted by the combination of the number of sacrificed lateral branches of the recipient artery, the number of sacrificed lateral branches with a diameter larger than 200 μm, and the clamp time with the minimal AIC value (AIC = 54.17). Among them, the clamp time was the only significant predictive factor [odds ratio, 1.087; 95% confidence interval (CI): 1.03–1.16, *p* = 0.0080; Table 2].

**Table 2:** Results of the logistic regression analysis for the predictive factors of the occurrence of anastomotic site infarctions (ASIs).

	**OR**	**95%CI**		***p*-value**
No. of sacrificed branches	1.399	0.944	2.336	0.1293
No. of sacrificed branches with the diameter larger than 200 μm	0.184	0.008	1.136	0.1491
Clamp time (min)	1.087	1.027	1.166	0.0080^*^

Forward and backward stepwise logistic regression analyses with AIC reveal that the occurrence of ASIs is predicted by the combination of the number of sacrificed lateral branches of the recipient artery, number of sacrificed lateral branches with a diameter larger than 200 μm, and clamp time with the minimal AIC value (AIC = 54.17). Among them, the clamp time is the only significant predictive factor (odds ratio, 1.087; 95% CI: 1.03–1.16, p = 0.0080). AIC = 54.17. ^*^Indicates statistically significant.

### 3.4. ROC analysis

**Figure 5:**
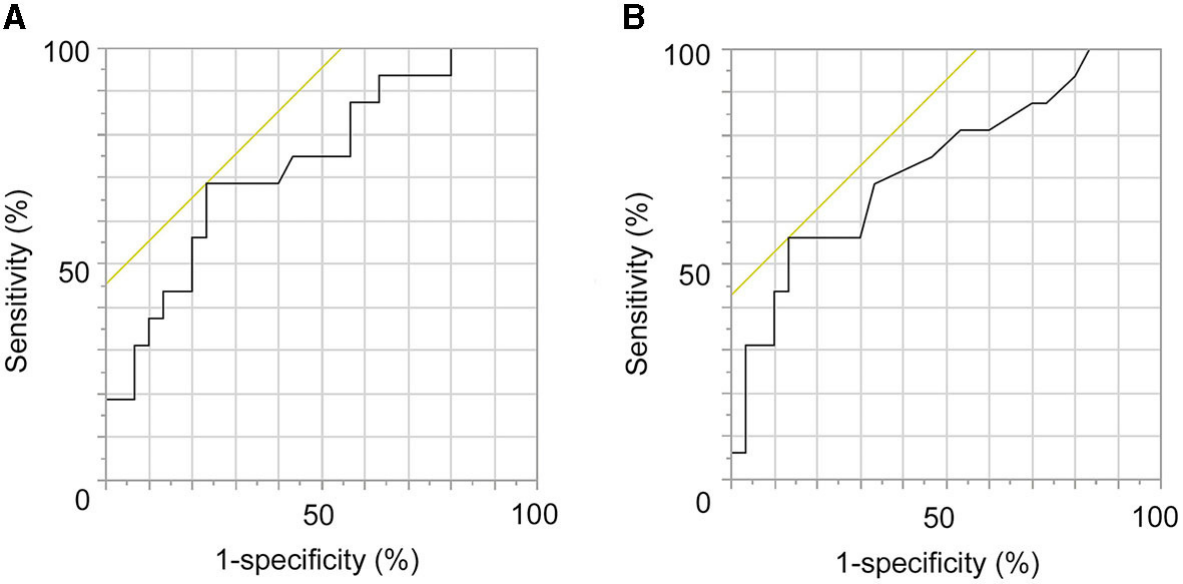
Receiver operating characteristic (ROC) analysis regarding the prediction of occurrence of anastomotic site infarctions (ASIs). **(A)** The clamp time is a significant predictive factor of the occurrence of ASIs (*p* = 0.0041; AUC = 0.7346; cutoff value, 33.2 min; sensitivity, 68.8%; specificity, 54.6%). **(B)** Clamp time per suture was a significant predictive factor of the occurrence of ASIs (*p* = 0.0047; AUC = 0.7271 cutoff value, 3.6 min; sensitivity, 58.3%; specificity, 57.1%).

The ROC analysis revealed that the clamp time was a significant predictive factor for the occurrence of ASI (*p* = 0.0041; AUC = 0.7346; cutoff value, 33.2 min; sensitivity, 68.8%; specificity, 54.6%; Figure 5A). Moreover, the clamp time per suture was a significant predictive factor for the occurrence of ASI (*p* = 0.0047; AUC = 0.7271 cutoff value, 3.6 min; sensitivity, 58.3%; specificity, 57.1%; Figure 5B).

## 4. Discussion

The relationship between the clamp time of the major cerebral artery and the occurrence of postoperative infarction has been examined mainly during aneurysm surgery. Many researchers have investigated the duration for which major cerebral arteries in aneurysm surgery, such as the M1 portion of the MCA, ICA, dominant A1 portion of the anterior cerebral artery, and basilar artery (BA), can be occluded without ischemic complications. The mean safe occlusion times of the ICA, M1, dominant A1, and BA were 28 (range, 27–29), 31 (range, 14–93), 40, and 15.5 min (range, 13–18), respectively (10–16). Other researchers have reported that the ischemic risk in high-flow bypass with temporary occlusion of proximal cerebral arteries longer than 10 min without pharmacologic brain protection would be ~45%. Even under brain protection, the ischemic risk remains 10–20%. This risk would increase to 100% when the occlusion lasts longer than 30 min. Therefore, the placement of an assisted bypass to the cortical artery distal to the recipient artery is recommended for high-flow bypass (17–19).

Many studies have reported the occurrence of postoperative symptomatic major infarction after STA-MCA anastomosis for MMD. The occurrence rates ranged from 4.7 to 21.4% (20–26). Ischemic onset (20), preoperative ischemic presentation (21, 23), frequent preoperative transient ischemic attack (TIA) (23), short intervals between the last ischemic attack and the operation (20), young age (25), old age (22, 25, 26), advanced Suzuki grade (21, 24), and posterior cerebral artery involvement (20, 24–26) were reported as significant predictive factors. The lack of strict perioperative management, including intraoperative normocapnia and normotension and postoperative normotension, were also significant predictive factors (20). However, the clamp time has never been considered a predictive factor for the occurrence of major stroke after surgery. In our study, postoperative symptomatic infarction occurred in two hemispheres (three anastomoses cases) among the 25 hemispheres (46 anastomoses cases; 8% per hemisphere; 6.5% per anastomosis), which was not significantly related to the clamp time (*p* = 0.9114).

It is unclear whether the clamp time in the M4 portion is related to the occurrence of ischemic complications after STA-MCA bypass for MMD. Horn et al. (8) reported that the occurrence rates of postoperative TIA and asymptomatic stroke on DWI after STA-MCA anastomosis with a clamp time of M4 ranging from 23 to 45 min were 10 and 10%, respectively.

DWI with 3T MRI can sometimes detect asymptomatic small ischemic lesions in the cortex near the anastomotic site after STA-MCA anastomosis. We defined such lesions as ASIs. Murai et al. suggested that ASIs are derived from the coagulation and sacrifice of the lateral cortical branches of the recipient artery. In our study, the multivariate analysis revealed that the clamp time of the M4 portion was the only significant predictive factor for the occurrence of ASIs. The number of sacrificed lateral branches and that of sacrificed large lateral branches were not significantly related to the occurrence. The ROC analysis revealed that the cutoff values regarding the clamp time and clamp time per suture were 33.2 and 3.6 min, respectively.

To reduce the clamp time, some modifications to the surgical technique and equipment have been reported. While we reported the “needle parking technique,” a modification of the conventional interrupted suturing to avoid needle loss during knot tying and to reduce the clamp time (27), Krisht et al. (28) reported a similar modification in 2020. Kohno et al. (29) reported the use of the scissors-attached micro-forceps that could significantly reduce the clamp time. The micro-forceps were 150 mm long and relatively large. We designed the scissors-attached micro-forceps suitable for MMD, which could significantly reduce the clamp time, clamp time per suture, and occurrence of ASIs.

### 4.1. Limitations

This study had some limitations. We conducted double anastomosis by using conventional micro-forceps for one anastomosis and scissors-attached micro-forceps for another anastomosis in the same operation on 17 hemispheres of 25 hemispheres in this study. Therefore, 33 anastomose of 46 anastomose in this study were performed using both micro-forceps in the same hemisphere in the same person. Approximately 75% of the patient clinical parameters in CG and SG were the same. That is because we avoided the logistic regression analysis regarding the prediction of ASI occurrence including patient clinical parameters in this study. In addition, it was a retrospective study with a relatively small sample size. Further prospective studies with more patients are required to confirm our results.

## 5. Conclusion

The newly developed scissors-attached micro-forceps could significantly reduce the clamp time and occurrence of ASI in STA-MCA anastomosis for MMD. The clamp time was the only significant factor predicting the occurrence of ASIs, with a cutoff value of 33.2 min.

## Data availability statement

The raw data supporting the conclusions of this article will be made available by the authors, without undue reservation.

## Ethics statement

The studies involving humans were approved by the Ethics Committee of Faculty of Medical Sciences, University of Fukui (No. 20150155). The studies were conducted in accordance with the local legislation and institutional requirements. The participants provided their written informed consent to participate in this study.

## Author contributions

MY: Writing—original draft, Data curation, Formal analysis, Methodology, Project administration, Validation, Writing—review and editing. HT: Data curation, Methodology, Project administration, Writing—original draft, Conceptualization, Supervision. RK: Writing—original draft, Conceptualization, Data curation, Resources, Software, Supervision. MI: Writing—original draft, Data curation, Methodology, Supervision. YH: Data curation, Methodology, Supervision, Writing—original draft. KM: Data curation, Methodology, Supervision, Writing—original draft. TY: Investigation, Software, Data curation, Writing—original draft. AA: Supervision, Data curation, Investigation, Writing—original draft. SK: Software, Data curation, Investigation, Writing—original draft. MO: Methodology, Supervision, Data curation, Writing—original draft. SY: Data curation, Methodology, Supervision, Writing—original draft. TT: Software, Data curation, Methodology, Writing—original draft. AW: Data curation, Methodology, Software, Writing—original draft. HO: Visualization, Data curation, Methodology, Writing—original draft. YK: Supervision, Validation, Data curation, Visualization, Writing—original draft. HA: Investigation, Methodology, Project administration, Data curation, Supervision, Writing—original draft. KK: Conceptualization, Formal Analysis, Funding acquisition, Resources, Software, Validation, Visualization, Writing—review and editing, Data curation, Investigation, Methodology, Project administration, Supervision, Writing—original draft.
